# Portraying the dark side of endogenous IFN-λ for promoting cancer progression and immunoevasion in pan-cancer

**DOI:** 10.1186/s12967-023-04453-4

**Published:** 2023-09-11

**Authors:** Zhen Zhen Wang, Xiao Ling Wen, Na Wang, Xu Hua Li, Yu Guo, Xu Zhu, Shu Heng Fu, Fei Fan Xiong, Jin Li, Limei Wang, Xiao Ling Gao, Hong Jiu Wang

**Affiliations:** 1 Key Laboratory of Tropical Translational Medicine of Ministry of Education, College of Biomedical Information and Engineering, Hainan Medical University, Haikou, People’s Republic of China; 2 College of Bioinformatics Science and Technology, Harbin Medical University, Harbin, People’s Republic of China; 3 The Medical Laboratory Center, Hainan General Hospital, Haikou, 570311 China

**Keywords:** Endogenous IFN-λ, Cancer-promoting, Multi-omics, Tumor microenviroment, Copy number variation

## Abstract

**Background:**

IFN-λ has been shown to have a dual function in cancer, with its tumor-suppressive roles being well-established. However, the potential existence of a negative ‘‘tumor-promoting’’ effect of endogenous IFN-λ is still not fully understood.

**Methods:**

We conducted a comprehensive review and analysis of the perturbation of IFN-λ genes across various cancer types. Correlation coefficients were utilized to examine the relationship between endogenous IFN-λ expression and clinical factors, immune cell infiltration, tumor microenvironment, and response to immunotherapy. Genes working together with IFN-λ were obtained by constructing the correlation-based network related to IFN-λ and the gene interaction network in the KEGG pathway and IFN-λ-related genes obtained from the networks were integrated as candidate markers for the prognosis model. We then applied univariate and multivariate COX regression models to select cancer-specific independent prognostic markers associated with IFN-λ and to investigate risk factors for these genes by survival analysis. Additionally, computational methods were used to analyze the transcriptome, copy number variations, genetic mutations, and methylation of IFN-λ-related patient groups.

**Result:**

Endogenous expression of IFN-λ has been linked to poor prognosis in cancer patients, with the genes IFN-λ2 and IFN-λ3 serving as independent prognostic markers. IFN-λ acts in conjunction with related genes such as STAT1, STAT2, and STAT3 to affect the JAK-STAT signaling pathway, which promotes tumor progression. Abnormalities in IFN-λ genes are associated with changes in immune checkpoints and immune cell infiltration, which in turn affects cancer- and immune-related pathways. While there is increased immune cell infiltration in patients with IFN-λ expression, this does not improve survival prognosis, as T-cell dysfunction and an inflammatory environment are also present. The amplification of IFNL2 and IFNL3 copy number variants drives specific endogenous expression of IFN-λ in patients, and those with this specific expression have been found to have more mutations in the TP53 gene and lower levels of DNA methylation.

**Conclusion:**

Our study integrated multi-omics data to provide a comprehensive insight into the dark side of endogenous IFN-λ, providing a fundamental resource for further discovery and therapeutic exploration in cancer.

**Supplementary Information:**

The online version contains supplementary material available at 10.1186/s12967-023-04453-4.

## Background

Type III IFN has been discovered recently and includes four IFN-λ genes of IFNL1, IFNL2, IFNL3 and IFNL4 in humans which are clustered on chromosome 19 [1, 2]. The potent antitumor activity of IFN-λ has been confirmed by independent groups in some tumor models. HCC827 cells with EGFR mutations treated with IFN-λ2 was reported to undergo growth inhibition and apoptotic cell death via STAT1 phosphorylation in a lung cancer model [3]. Moreover, IFN-λ induced G1 phase arrest or apoptosis in esophageal cancer cells and produced antitumor effects in combination with anti-cancer drugs [4]. In addition, indirect antitumor effects of IFN-λ have also been widely reported, including immune cell activation and angiogenesis inhibition. The IFN-λ plays a role in inhibiting tumor angiogenesis in a primary B16 mouse melanoma model, suggesting that type III IFN is involved in a host mechanism that inhibits melanoma growth [5]. Usp18 deficiency in mammary epithelial cells produces an anti-tumor environment caused by a hypersensitive response to IFN-λ and elevated Cxcl10 secretion [6].

Despite the potent antitumor activity of IFN-λ, it also exhibits a dual role in cancer, and has been shown that IFN-λ induces tumor cell migration and tubule formation. As shown in a bladder cancer model IFN-λ induces matrix metalloproteinase 9 expression and promotes tumor migration and invasion, as well as the development of bladder cancer associated with disease progression [7]. Induction of cancer metastasis by bone marrow-derived suppressor cells through IFN-λ production has been demonstrated [8, 9]. In addition, IFN-λ promotes angiogenesis, epithelial-mesenchymal transfer, invasion and migration of tumor cells in a STAT3-dependent manner. Hence, although IFN-λ has a tumor suppressive effect, probably during late cancer stages, IFN-λ also acts as a cancer promotor, suggesting that IFN-λ can also promote carcinogenesis, highlighting the urgent need to further identify the dark side of IFN-λ in cancer and explore clinically useful biomarker tests to facilitate and improve patient monitoring and treatment. However, there are few studies on the negative role of IFN-λ in pan-cancer.

In this study, we performed a systematic analysis of the dark side of IFN-λ in tumors by integrating multi-omics and ligand/receptor data for pan-cancer patients. This reveals that IFN-λ was specifically expressed and associated with poor patient prognosis. In addition, IFN-λ acted together with related genes such as STAT1, STAT2, and STAT3 affecting the JAK-STAT signaling pathway to promote cancer progression. Further analysis demonstrated that patients with specific expression of IFN-λ had T-cell dysfunction, a stronger inflammatory environment and immune cell infiltration. Moreover, IFN-λ specific expression may be caused by copy number amplification of IFNL2 and IFNL3 in tumor patients and driven cancer progression. In conclusion, we integrated multi-omics data, systematically combined with the analysis of pan-cancer patients, to reveal complex aspects of IFN-λ in its tumor promotion role.

## Methods

### Molecular and clinical information of the tumor datasets

Multi-omics data were downloaded from TCGA. The gene expression data of 9398 samples for 32 cancers were downloaded from UCSC Xena (https://xenabrowser.net/), with FPKM-UQ normalization. The copy number variation (CNV) data were obtained from UCSC Xena for 22,445 genes. The CNV data for TCGA samples included four non-diploid normal copy states (− 2), single copy deletion (low copy number 1), low copy number amplification (1) and high copy number amplification (2). The somatic mutation data was acquired from TCGA database.

The DNA methylation 450 k data of pan-cancer were downloaded from UCSC Xena. The data showed the DNA methylation values (β value) of each array probe in each sample. The DNA methylation value is a continuous variable between 0 and 1, indicating the degree of methylation. Higherβvalue indicate hypermethylation, and lowerβvalue indicate hypomethylation.

The clinical data of TCGA samples including gender, age, tumor weight, TNM stage, survival time were downloaded by the GDC tool (https://portal.gdc.cancer.gov/). All datasets were partitioned into training and testing sets with a ratio of 80%, 20%, respectively.

### Consensus clustering

We performed unsupervised clustering of patients, using consensus clustering based on cancer-specific expression of ligand or receptor genes in TCGA cohort. ConsensusClusterPlus [10] R-package was used to identify the structure and relationship of the patients with the following algorithms: hierarchical clustering with agglomerative ward linkage (HC), K-means on a distance matrix (KMdist), and partitioning around medoids (PAM) and clustering measures with Pearson correlation (Pearson), Spearman correlation (Spearman), and Euclidean distance (Euc), Manhattan distance (Manhattan), Binary correlation (Binary) as the dissimilarity measure. Using consensus clustering with resampling (10,000 iterations) with the number of clusters ranging from 2 to 10, pFeature = 1, representing = 50, pItem = 0.8, we compared the consistency of cluster results on different cluster algorithms and measure. The final optimal number of clusters is determined by calculating the proportional increase of the area under the number of the cumulative density function (CDF) curves, which plots the corresponding empirical cumulative distribution defined in the range of 0 to 1, and then the optimal cluster is determined.

### Correlation analysis of IFN-λ expression in TME

For the datasets in this study, we evaluated their cell abundance profiles by used DCNet including 106 immune cells, 322 stromal cells, and 6 cancer cells [11]. To characterize the cell states associated with patients expressing endogenous IFN-λ, 14 cell states and their signature genes were obtained from the CancerSEA. We defined the cell state score to evaluate TME of patients and the cell state scores were calculated by using the GSVA R package, which initially depended on the variation of gene set enrichment from the predefined signature gene set.

### Analysis of genomic mutation and methylation profile

The R package ‘ConsensusClusterPlus’ (version 1.54.0) was used to perform consistent clustering on Tumor mutation burden (TMB) for each patient can be obtained using the VarScan [12] method, as calculated by the R package "mafools" [13]. The annotation information of the methylation sites was positioned onto the coordinates of the human genome using the GEO GPL13534 derived xena probeMap for microarray probes, including cpg, gene location, base changes, chromosomes, etc. We compared the differential methylation probes (DMPs) based on t-test. The absolute value of methylation difference greater than 0.15 and a p-value less than 0.05 was considered to be differentially methylated. Differentially methylated genes were identified using the limma package. R package clusterProfiler was performed for GO analyses of the DEGs and DMP-related genes and Gene set enrichment analysis (GSEA) [14].

### Establishing and evaluating the prognosis prediction model

Univariate Cox regression analysis was used to evaluate the relationship between the expression of various differentially expressed genes and the overall survival (OS) of patients. The various significant univariables were further subjected to multivariate analysis to construct a prognostic risk model. The risk scores for each samples were established using the "prediction" function in the survival package as follows:

$${\text{RiskScore}} = \sum \beta {\text{i }} \times {\text{Mi}}$$

$\beta {\text{i}}$ is the risk regression coefficient of the multiple Cox analysis of each gene, and ${\text{Mi}}$ is the gene expression value. All samples were divided into high and low-risk groups according to the median risk value of each sample.

### Identify differentially expressed genes

Differentially expressed genes (DEG) analysis was performed using the R package “limma” [15], The DEGs were identified with false discovery rate (FDR) < 0.05 and |log2FoldChange|> 2.

### Constructing the patient-specific IFN-λ co-expressed ligand-receptor network

Based on the ligand-receptor networks obtained from CellTalkDB database (http://tcm.zju.edu.cn/celltalkdb/index.php), we first constructed the relevant IFN-λ ligand-receptor networks with the IFNL2 and IFNL3 genes. Subsequently, the significant elevated levels of ligand or receptor gene expression from NSE to BSE group were selected. The co-expression of genes was considered with a threshold of coefficient > 0.2 and by applying the "p.adjust" function to calculate the adjusted P-value in R, with a p-value < 0.05. Thus, the co-expressed ligand-receptor network related with IFN-λ was constructed based on the retention of the co-expressed ligand-receptor pairs.

### Protein–protein interaction network analysis

The protein–protein interaction network (PPI) of the target genes (STAT1, STAT2 and STAT3) was obtained from the STRING [16] database (https://string-db.org/) with a minimum required interaction score of 0.4 and a PPI enrichment of p < 1.0e−16. In the PPI network, the nodes represent the target protein, while the edges represent the predicted or validated interactions between the proteins. Subsequently, in order to construct the IFN−λ associated protein network, we used Pearson correlation analysis of the edges to obtain interactions, retaining correlation values greater than 0.2 edges. Then, the nodes with significantly higher gene expression levels from the BSE to the NSE group were retained (Wilcoxon test p < 0.05). The IFN−λ associated protein network was visualized in Cytoscape software (version 3.7.1, www.cytoscape.org).

### Statistical analysis

Comparisons with different variance were analyzed using the Wilcoxon test. Correlations between the two variables were mesasured using the Pearson’s correlation coefficient, and the corresponding significance was evaluated using a two-sided hypothesis test. Survival analysis was performed using the Kaplan–Meier (KM) method, with the R package ‘‘survival’’ and ‘‘survminer’’ to calculate survival differences among patient groups. Based on the expression value of the IFN-λ related genes, we performed unsupervised learning on the training set using the Random Forest R package. Finally, we used the R package pROC to evaluate the ROC curve and the AUC for each model [17]. Functional annotation analysis of GO and KEGG revealed biological functions using the R package "clusterProfiler" [18]. All P-values were two-sided, and P < 0.05 was considered to indicate a statistically significant difference.

## Results

### IFN-λ genes were identified as tumor-specific expression receptors and associated with patients poor prognosis

**Fig. 1:**
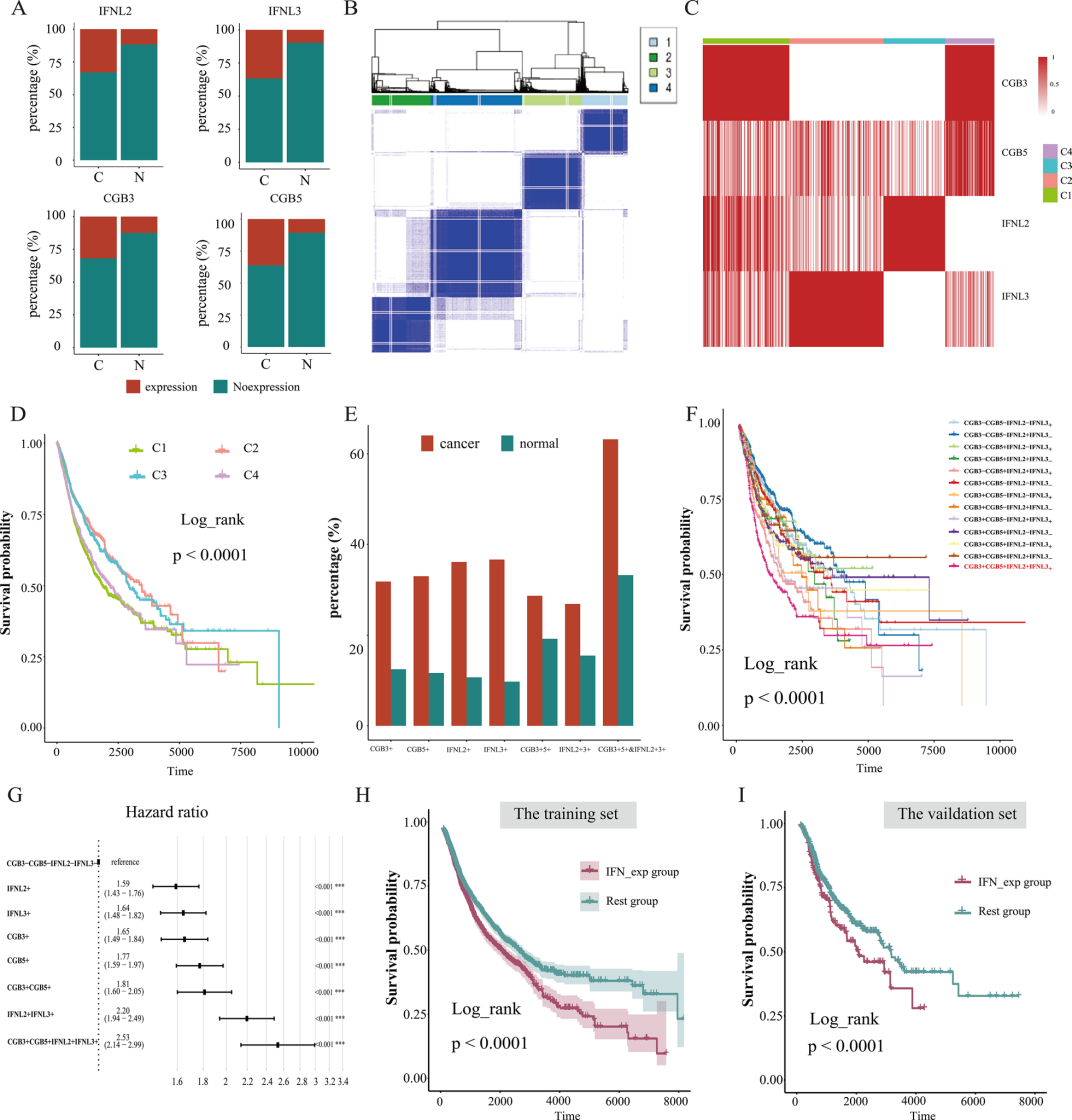
**A** Bar plots showing proportions of IFNL2, IFNL3, CGB3 and CGB5 specific expression in cancer and normal samples. C, cancer, N, normal. **B** Consistent clustering result by the K-means method to cluster the TCGA training set into four groups. **C** Heatmap showed the expression of the specific-genes in four groups. **D** Kaplan–Meier curves of OS among the four patient groups in the training cohort. **E** The histogram showing proportions of the gene specific expression incancer and normal samples. -, no-expression, + , expression **F** Survival analysis for subgroups patients stratified by GSE using the Kaplan–Meier curves. **G** Forest plot of multivariate Cox regression analysis for GSE subgroups related to prognosis. **H**–**I** Kaplan–Meier estimates of overall survival for the IFN_exp and the rest group in the training **H** and validation **I** cohort

To investigate whether there exist expression-specific ligand and receptor genes in cancers, which may be affect tumor progression and prognosis. We collected 780 ligands and 815 receptors genes information from the CellTalkDB database and investigated differences in the proportion of ligand or receptor gene-specific expression in tumor and normal tissues, and then just four genes of IFNL2, IFNL3, CGB3, CGB5 demonstrated tumor-specific expression in more than 30% of all cancer cases (3394, 3444, 2993, 3098 samples, respectively), while only in a small amount of normal cases(78, 71, 91, 85 samples, respectively, Fig. 1A). Then, using Consensus clustering of multiple K-means with resampled the four specifically expressed profiles (Additional file 1: Figure S1A–B), the patients were partitioned into four groups temporally named as C1 to C4, and four genes’ specific expression pattern showed dependent-specific expression patterns in the four patient groups (Fig. 1B). IFNL2 was mainly specifically expressed in C3 patient group, and IFNL3 was specifically expressed in C2 patient group (Fig. 1C). The CGB3 and CGB5 genes were specifically expressed in C1 and C4 patient group, respectively. These findings suggest that specific expression of the four genes occurs in specific populations. Moreover, Kaplan–Meier survival analysis showed that there were significant differences in survival among those four patient groups, indicating that these genes specific expression (GSE) might affect patient outcomes (p < 0.001, Fig. 1D). We also compared the distribution of these GSE between tumor and normal patients, and it showed that tumor patients tended to carry more GSE, suggesting that those genes’ expression may relate to tumor aggressiveness (Fig. 1E).

Next, to analyze the survival value of patients with these specific expression genes, we partitioned patients into 13 groups according GSE. Prognosis analysis of 13 patient groups revealed the patients with specific expression of all four gene had a survival disadvantage (Log-rank test P < 0.0001, Fig. 1F). Interestingly, we also found a trend of poorer survival in patients carrying both IFNL2 and IFNL3 genes expressed. Multivariate cox regression analysis showed that patients with IFNL2 + IFNL3 + was associated with worse clinical outcomes, independent of age, sex, and stage status (IFNL2 + IFNL3 + , HR = 2.20, p < 0.001, Fig. 1G). These findings showed that IFNL2 and IFNL3 were dominant risk factor for overall survival. IFNL2 and IFNL3 had been reported to be the type III interferon genes (IFN-λ) and IFN-λ had a dual role in cancer. Our results suggest that the endogenous IFN-λ can promote tumorigenesis, providing support for subsequent studies. Subsequently, we focused the research on the patients with the specific expression of IFNL2 and IFNL3 genes, and defined the patient population with IFN-λ (IFNL2 and IFNL3) specific expression as the IFN_exp group and the rest population as the rest group. The survival analysis showed that the IFN-λ specific expression was associated with a poor prognosis in the training cohort, which also was validated in the validation dataset (p < 0.05, Fig. 1H, I). Collectively, we revealed that IFN-λ genes (IFNL2/IFNL3) specific expression status may be a potential prognostic biomarkers, which deserves to be further explored.

### Prognostic impact of the expression of the patients' endogenous IFN-λ

**Fig. 2:**
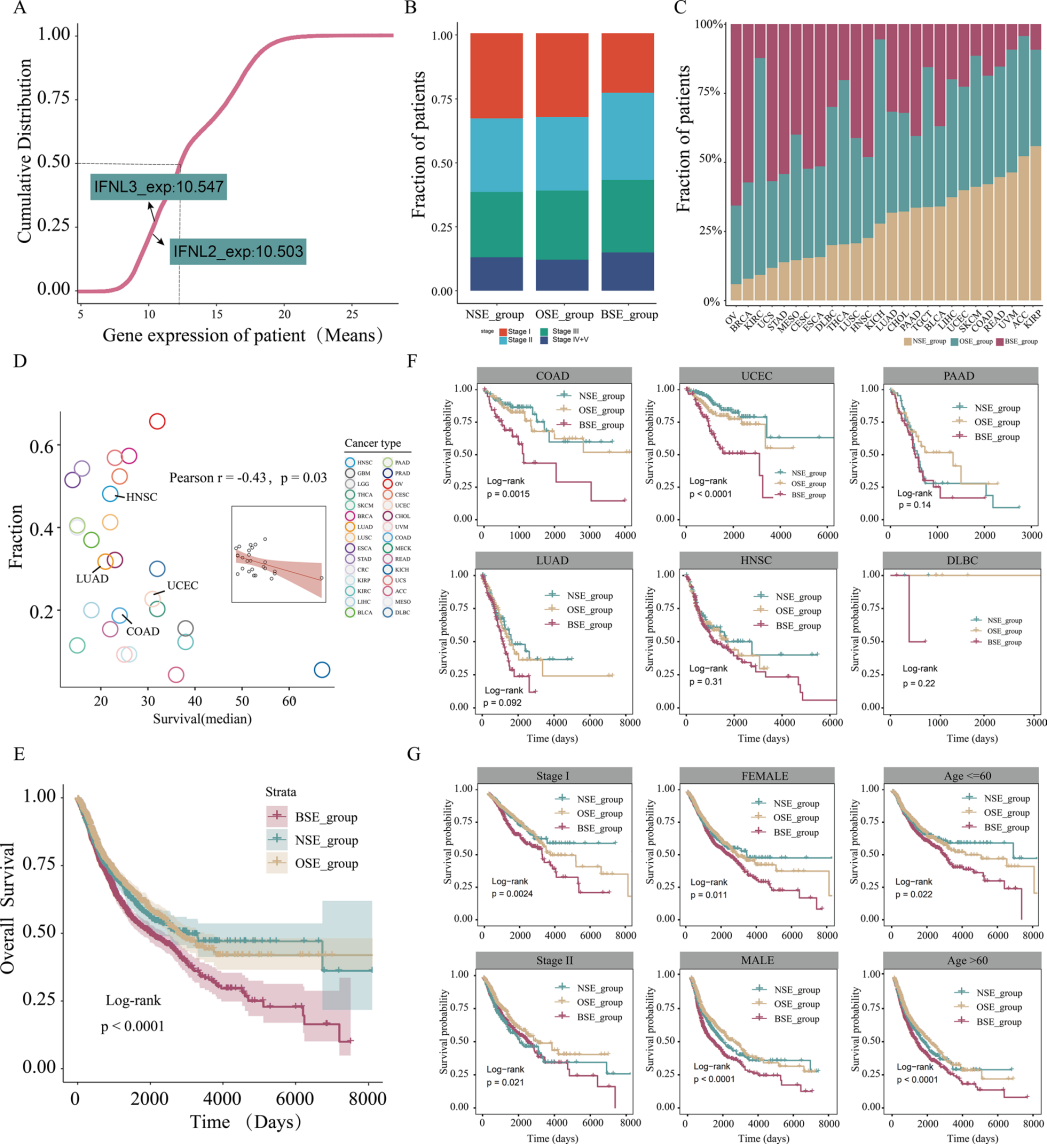
**A** Cumulative distribution map of genes expression in pan-cancer. **B** Distribution of pathological stages among the three IFN-λ subgroups. **C** Distribution of the three IFN-λ subgroups among the different tumor types. **D** Correlation between number of patients with IFN-λ expression and median survival across cancer types in BSE_group. **E** Relationship between each IFN-λ subgroup and OS in pan-cancer. **F** Kaplan–Meier survival plot in several representative tumor types. **G** Kaplan–Meier survival analysis of each IFN-λ subgroup at each cancer stage, gender and age subgroup

To examine the expression levels of the endogenous IFN-λ, we compared the average expression of IFN-λ among all human genome genes and found that endogenous IFN-λ exhibited a relatively low level of expression in cancers (IFNL2, 10.503, IFNL3, 10.547 and all genes, 13.38, Fig. 2A). We continued to investigate the effects of endogenous IFN-λ expression on the clinical outcome. Based on the IFNL2/IFNL3 gene was specifically expressed or not expressed, patients were partitioned into three IFN-λ subgroups (NSE_group: patients without IFNL2/IFNL3 specific expression, OSE_group: patients with IFNL2 or IFNL3 specific expression, BSE_group: patients with IFNL2 and IFNL3 specific expression). The BSE group has more patients with advanced, suggesting that patients with endogenous IFN-λ expression may have a poor prognosis, which is consistent with our previously obtained results of Figs. 1H, 2B). Next, we examined whether there are significant differences in the proportion of patients with IFN-λ expression among different cancer types, and it revealed that individual solid tumor types varied substantially in their composition of IFN-λ subgroups (Fig. 2C). Meanwhile, we found that those cancer types were negatively correlated with survival times (Pearson test r = − 0.4, p < 0.05, Fig. 2D), suggesting the patients with carrying more expression of endogenous IFN-λ may have a poorer prognosis.

Afterward, to explore the survival value of the three IFN-λ subgroups in pan-cancer, we performed Kaplan–Meier analysis, which showed that BSE patients were associated with poor prognosis, whereas NSE patients were associated with superior prognosis (Log-rank test p < 0.0001, Fig. 2E). Subsequently, we further explored whether IFN-λ could be used as a predictor of cancer survival. We performed cancer type-specific survival analysis also revealed significant associations between the IFN-λ subgroups and OS in a number of cancer types. As shown in Fig. 2F, patients with BSE appeared to be associated with poor prognosis in COAD, UCEC, PAAD, LUAD, HNSC and DLBC. These results indicating that IFN-λ can serve as a predictor for survival in most cancers. Next, we further examined whether the poor prognosis in patients with IFN-λ expression would be influenced by clinicopathological variables. We performed survival analysis after partitioning into specific subgroups according to different clinical features and found that the association between the IFN-λ subgroups and OS remained significant (p < 0.001, Fig. 2G), indicating that IFN-λ can serve as an independent predictor for survival prediction. Overall, endogenous IFN-λ expression are associated with a poor prognosis in most cancers, and it can also serve as an independent predictor of outcomes. These results suggest that the tumor pathogenesis is closely associated with the endogenous expression of IFN-λ, and an existence of a dark ‘‘tumor-promoting’’ side effect of IFN-λ.

### IFN-λ and its related genes work together to promote cancer progression

To further understand the dark side of endogenous IFN-λ for promoting cancer progression, we systematically analyzed the IFN-λ-related genes expression changes from the angles of receptor-ligand interaction (RLIs), protein–protein interaction (PPI) and transcriptional regulation.

**Fig. 3:**
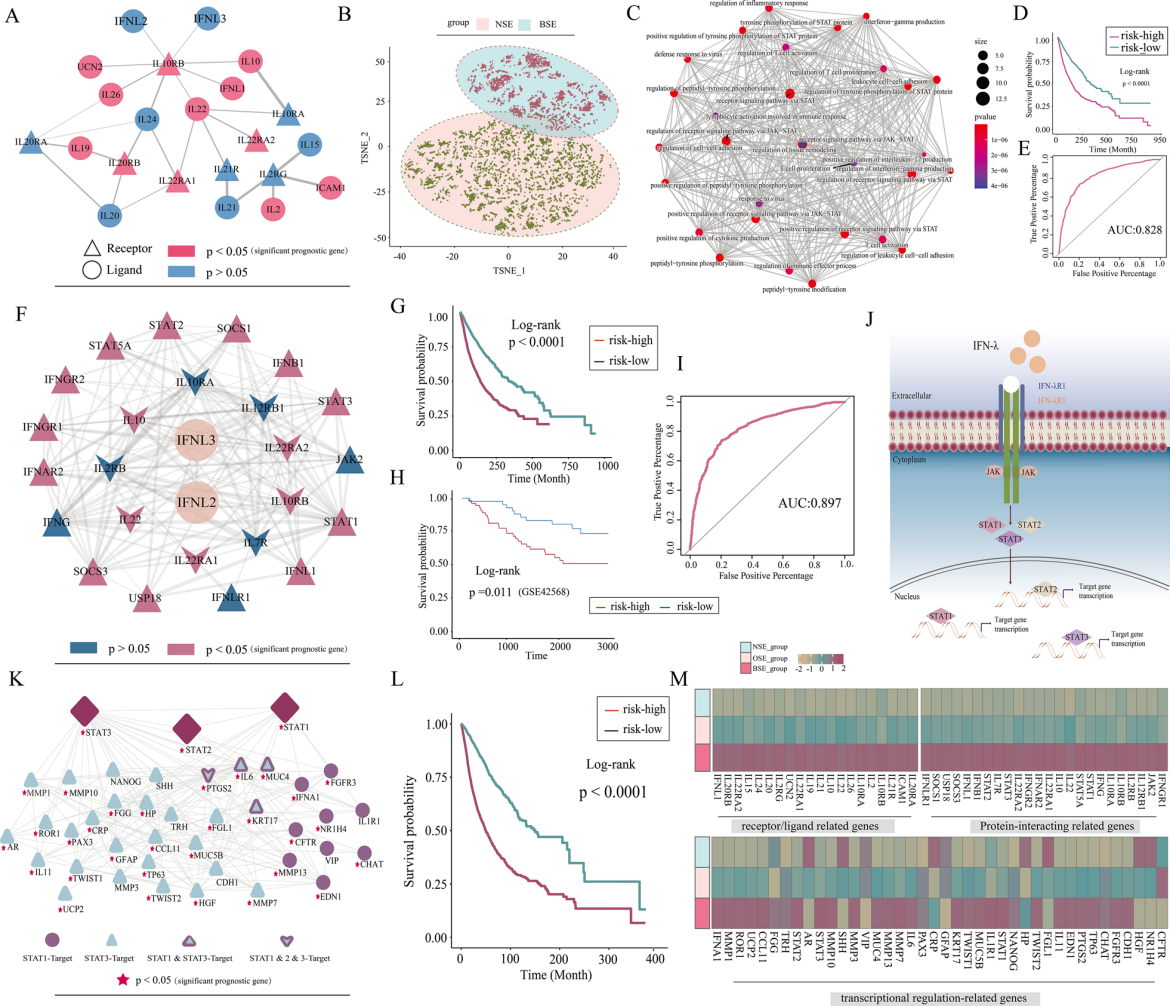
**A** The ligand-receptor network of IFN-λ-related gene. **B** Analysis of the t-SNE of the pan-cancer samples based on the expression of ligand-receptor genes, the NSE group patients, pink; the BSE group patients, blue. **C** Interaction of the enriched pathways. The size represents the number of genes, and the color represents the p-value. **D** KM survival curve for classifying the high and low risks of TCGA validation set samples was constructed by IFN-λ-related ligand receptor genes as features. **E** The ROC curve of the model. **F** The PPI network of IFN-λ-related gene. **G** KM survival curve for classifying the high and low risks of TCGA validation set samples was constructed by IFN-λ- related proteins genes as features. **H** KM survival curves of OS of patients in the low- and high-risk groups in GSE42568 cohort. **I** The ROC curve of the model. **J** Pictorial representation of IFN-λ regulation combined our findings. **K** Diagram of the transcription factors (STAT1, STAT2, STAT3) and the target gene networks. **L** KM survival curve for classifying the high and low risks of TCGA validation set samples was constructed by IFN-λ- related target gene as features. **M** The IFN-λ associated genes normalized expression in the three groups

Firstly, we examined the significant upregulated expression of IFNL2/IFNL3-related receptor or ligand genes among three IFN-λ subgroups and identified 20 IFN-λ related receptor or ligand genes (R > 0.15, p < 0.05, Fig. 3A). Furthermore, we performed principal component analysis (PCA) of the 20 gene expression matrix showing that patients from the BSE and NSE groups were clearly separated into two distinct groups, suggesting a high degree of concordance between these genes expression changes and IFN-λ (Fig. 3B). We then further examined the biological functions of these genes consistent with IFN-λ expression to see if they affect cancer development from other perspectives. The results showed that these 20 genes were associated with regulation of the inflammatory response, JAK-STAT signaling pathway, and cell–cell adhesion (Fig. 3C). We found that inflammation-related genes IL15, IL2, IL21, IL20, IL20, IL22RA2, IL20RB, and IL10 associated with IFN-λ expression promote inflammatory response signaling, suggesting that patients with endogenous IFN-λ expression may enhance the inflammatory environment and response in tumor lesions due to the upregulated expression of these genes. Interestingly, the IFN-λ expression activated the JAK/STAT signaling pathway through the upregulation of IL24, IL10, IFNL1, IL26, IL10RB, and IFNL1 expression, which promoted cell proliferation and tumorigenesis. Since the expression changes of 20 genes followed the same trend as IFN-λ expression changes, we further tested whether those genes expression can serve as independent prognostic markers, just like IFN-λ. Hence, we performed a univariate Cox regression analysis to identify significant prognostic genes (p < 0.05, Fig. 3A), including ICAM1, IL10RB, IL10, IL22RA, UCN2, IL20RB, IL2, IL26 and IL19, which were further used to establish multivariate Cox proportional risk regression models. Kaplan–Meier survival analysis showed results consistent with survival in the IFN-λ subgroups (p < 0.0001, Fig. 3D), indicating that these 13 genes could serve as an independent prognostic factor. Furthermore, we constructed a classifier based on the expression of these 13 genes to predict whether patients expressed endogenous IFN-λ using a random forest approach method (AUC, 0.828, Fig. 3E). The results suggested that these 13 genes have a good classification effect for patients who express IFN-λ or not.

Next, we continued to explore the negative effects of IFN-λ from perspective of protein interaction. We constructed a network of IFN-λ-related proteins that were significantly upregulated in the BSE group by integrating the expression information of IFN-λ associated interacting proteins from STRING databases (Fig. 3F). Many well-known proteins that play crucial roles in cancer like IL10, USP18, and IL22 were included in this network. In addition, we found that endogenous IFN-λ expression promoted the activation of JAK upon cytokine attachment and stimulated the phosphorylation of STATs in the intracellular region of the receptor to enhance the JAK-STAT pathway dysregulation that may lead to various immune disorders (Fig. 3F, J, Additional file 2: Figure S2A). Subsequently, we adopted the same strategy as mentioned above to test whether these IFN-λ-related proteins expression can serve as independent prognostic markers, just like IFN-λ, resulting in a multivariate Cox proportional risk regression model based on 17 IFN-λ associated genes (p < 0.05, Fig. 3F). Kaplan–Meier survival analysis showed poorer overall survival in the high-risk cohort compared to the low-risk cohort ( p < 0.0001, Fig. 3G), suggesting that the expression of these genes can be used as prognostic marker like IFN-λ. Moreover, the prognostic value of the model was validated in the GSE42568 cohort (Log-rank test p < 0.05, Fig. 3H). Next, we constructed a random forest classifier based on 17 genes as signature genes to examine whether their expression could predict patients with or without endogenous IFN-λ expression. The results showed an AUC value of 0.897, indicating that these genes can be used to assessed in patients with or without endogenous IFN-λ expression (Fig. 3I). These findings indicated that the expression changes of IFN-λ-related protein genes are consistent with IFN-λ, and the prognostic risk of patients can be accurately identified based on the expression of these genes.

According to the above results, the JAK-STAT pathway was significantly dysregulated in the production of endogenous IFN-λ expression, and it caused the upregulation of the transcription factors STAT1, STAT2, and STAT3 (Fig. 3J). Subsequently, we further explored the negative effects of IFN-λ from perspective of transcriptional regulation. We retrieved potential target genes for transcription factors including STAT1, STAT2, STAT3, using the TRRUST database to explore which downstream are affected and then identified 40 significantly different target genes among the IFN-λ subgroups using the R package limma. The relationship among the 40 target genes was visualized using Cytoscape (Fig. 3K). We found that upregulation expression of STAT3 target gene IL6 was influenced by endogenous IFN-λ expression, increasing the activation of the IL-6/JAK/STAT3 pathway which was abnormally hyperactivated in many types of cancer, and this hyperactivation is generally associated with a poor clinical prognosis [19]. Meanwhile, we noted that Matrix Metalloproteases (MMPs), including MMP1, MMP3, MMP7, MMP10 and MMP13 genes were significantly upregulated in the BSE group, and they were almost universally upregulated in cancer [20]. Then, we adopted the same strategy as mentioned above to test whether these target genes that IFN- λ affects downstream expression can serve as independent prognostic markers, just like IFN-λ, resulting in a multivariate Cox proportional risk regression model based on 35 target genes (p < 0.05, Fig. 3K). Kaplan–Meier survival analysis showed poorer overall survival in the high-risk cohort compared to the low-risk cohort (Log-rank test p < 0.0001, Fig. 3L), suggesting that these target genes affected downstream by IFN-λ expression can be independent of prognostic markers.

Overall, the above analysis identified significantly upregulated expression of IFN-λ related genes from different levels in BSE patients (Fig. 3M). Meanwhile, these IFN-λ-related genes and IFN-λ work together to influence the inflammatory response and JAK-STAT pathway to promote cancer progression, and that related-genes of each level like IFN-λ can act as independent prognostic risk factors.

### IFN-λ cancer-specific expression may be caused by copy number amplification of IFNL2 and IFNL3 in tumor patients

**Fig. 4:**
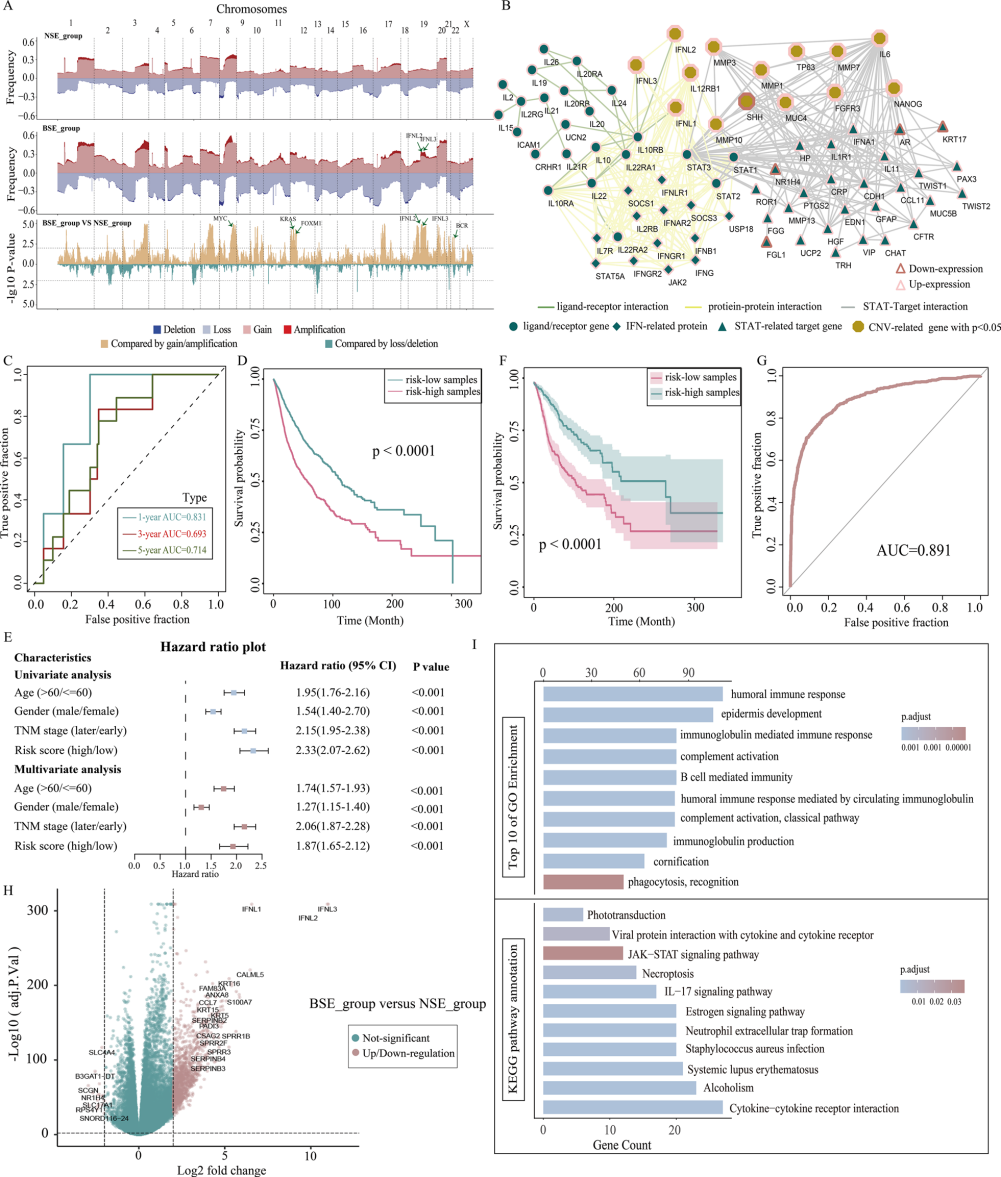
**A** Somatic CNA frequency of individual genes in each group plotted along the chromosomes (upper), comparisons of somatic CNA between BSE and NSE groups with − log10 FDR (Fishers exact test, lower). **B** The significant copy number variation affects the IFN-λ related network. **C** ROC curves of the risk score for predicting 1 year, 3 year, and 5 year survival. **D** Kaplan–Meier survival curves of OS of patients in the low- and high-risk groups in the training set. **E** Univariate and multivariate analyses of the clinical characteristics and risk score with the OS. **F** Kaplan–Meier showed the independent relevance between overall survival time and risk scores in the training set. **G** The ROC curve of the model. **H** Volcano plot showing the differentially upregulated and downregulated genes. **I** Bar plot showing the results of GO enrichment and KEGG by the differentially genes

Since our previous analysis revealed that IFN-λ specific expression occurred in some cancer patients with poor prognosis, we further explored the causes of IFN-λ cancer-specific expression from the perspective of genomic variation. We compared genomic alterations between the BSE and NSE groups with the CNV data obtained from UCSC Xena for TCGA pan-cancer patients. Genome-wide CNV revealed that the BSE patients had a significantly higher copy number variation, especially on chromosomes 3, 8, and 19, as shown in Fig. 4A. We further examined the detailed characterization of CNV across two groups. Between two groups, the differences of all genes in copy number amplification and deletion (− log10 FDR value) were calculated using Fisher’s exact test and found the BSE patients had more frequent somatic copy-number alterations (FDR < 0.01, Fig. 4A). In detail, the IFNL2 and IFNL3 were significantly amplified in the BSE group than the NSE group, and some representative oncogenes such as FOXM1, BCR, KRAS, MYC were widely amplified. These results suggest that significant amplification of the IFNL2 and IFNL3 may cause IFN-λ cancer-specific expression and that the BSE patients exhibited significant copy number amplification across the whole genome. Combined with IFN-λ-associated genes, we found that there were twelve genes, namely MMP3, IL2RB1, IFNL1, MMP10, SHH, MMP1, TP63, MMP7, MUC4, FGFR3, IL6, NANOG (− log10 FDR > 2), which had significant differences in the copy number between the BSE and NSE groups (Fig. 4A, B). These genes were more highly expressed in the BSE group (except for SHH) compared with the NSE group. The results indicate that IFN-λ-related genes undergo copy number amplification, which affects their expression on the transcriptome and acts in conjunction with IFN-λ to promote tumor development.

Subsequently, we further explored whether the expression levels of these twelve genes would affect the patient's prognosis. We then performed univariate Cox regression analysis to identify 11 significant prognostic genes (except for IL2RB1) in the training set (p < 0.05), which were used to build a multivariate Cox proportional hazards regression model. The risk score for each sample was calculated based on the established models, which has a high discriminatory power for survival status. The average AUC values for 1-year, 3 year, and 5 year outcome predictions on the training set reached 0.831,0.693, and 0.714 (Fig. 4C). Kaplan–Meier survival analysis indicated the high-risk group had a poorer overall survival compared with the low-risk group in the training (p < 0.001, Fig. 4D) and validation sets(p < 0.001, Fig. 4F). The results suggested that the expression levels of copy number amplified genes affect the prognosis of patients. Then, to determine the independence of the risk score in prognostic diagnosis. We performed univariate and multivariate Cox regression analyses to evaluate whether the risk model of 11 genes had independent prognostic characteristics for cancer patients. We found that OS of patients were significantly correlated with the prognostic model in the training (univariate Cox regression, HR = 2.33, 95% CI 2.07–2.62, p < 0.001; multivariate Cox regression: HR = 1.87, 95% CI 1.65–2.12, p < 0.001, Fig. 4E) and validation cohort (univariate Cox regression, HR = 2.04, 95% CI 1.64–2.54, p < 0.001; multivariate Cox regression: HR = 1.89, 95% CI 1.50–2.39, p < 0.001 Additional file 3: Figure S3A). The above results indicate that the model has good predictive power for the clinical outcome of patients. Next, we developed a random forest model to predict patients with and without IFN-λ expression by using these 11 prognostic genes (AUC, 0.891, Fig. 4G), indicating that these genes with copy number amplification have good identification of patients with and without IFN-λ expression.

Moreover, to further investigate the underlying biological behavior of IFN-λ, we identified 1,195 DEG between the BSE and NSE groups using limma R package, with 1,178 up-regulated and 17 down-regulated genes in the BSE group (fold change |log2FC |> 2, p < 0.01, Fig. 4H). Then, functional enrichment analyses using clusterProfiler were performed to infer the potential functions of these DEGs. Thees DEGs were found to be enriched in immune-related biological processes such as humoral immune response, epidermis development, and B cell mediated immunity (Fig. 4I), indicating that they have a positive role in the enhancement of tumor-associated immunity. Some of the upregulated genes such as CXCL13 [21] and CXCL11 [22] and KLK5 [23] have been experimentally verified in prior studies to regulate humoral immune response and support tumor infiltration. Furthermore, KEGG pathway enrichment analysis showed that these DEGs were mainly enriched in cytokine-cytokine receptor interactions and JAK-STAT signaling pathway pathways (Fig. 4I).

Overall, our analysis showed that IFN-λ cancer-specific expression may be caused by copy number amplification of IFNL2 and IFNL3 in tumor patients, and most IFN-λ related genes also had copy number amplification and expression upregulation in BSE group. Dysregulation of downstream related pathways caused by endogenous IFN-λ expression, including cytokine-cytokine receptor interactions, humoral immune responses and JAK-STAT signaling pathways, which may be drivers of cancer development.

### Patients with endogenous IFN-λ expression show unique tumor microenvironments

**Fig. 5:**
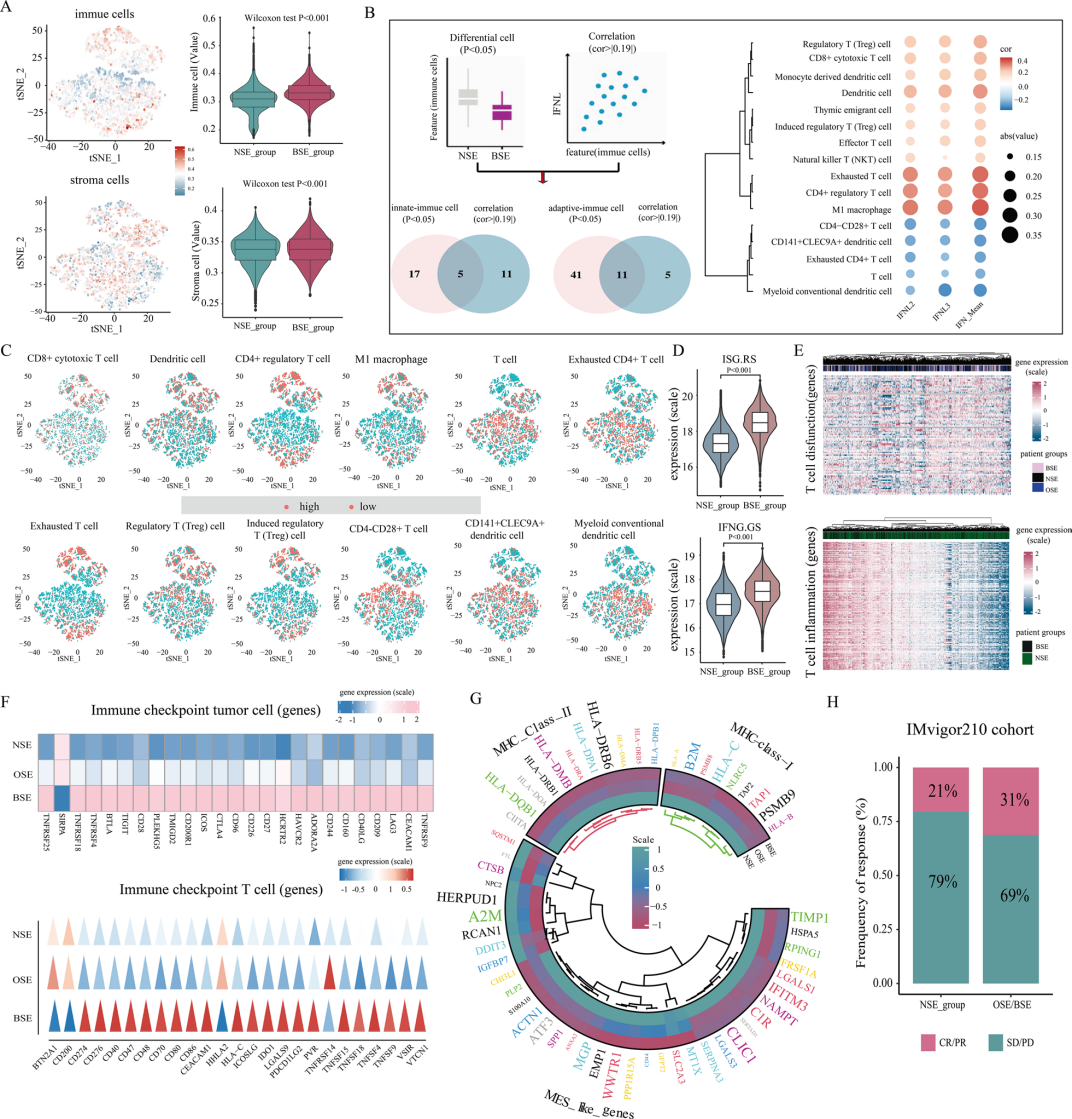
**A** Immune and stromal cell infiltration levels between BSE and NSE groups (left) and differences in immune and stromal cell infiltration between BSE and NSE groups (right). **B** Correlation between each infiltration cell type and IFN-λ expression. **C** Distribution of expression changes in immune cells. **D** Boxplots of resistance-associated ISGs (ISG.RS) and IFNG-related ISGs (IFNG.GS) expression in BSE and NSE groups. **E** Expression heatmap of T cell dysfunction and T cell inflammation related-genes. **F** Immune checkpoint tumor cell (upper) and immune checkpoint T cell (lower) corresponding gene normalized expression in three IFN-λ expression subgroups. **G** Heatmap showing the expression of major histocompatibility complex class I (MHC-I), MHC-II, and mesenchymal-like (MES-like) related genes in three IFN-λ expression subgroups. **H** The proportion of patients with response to PD-L1 blockade immunotherapy in NSE and BSE/OSE groups. *SD* stable disease; *PD* progressive disease; *CR* complete response; *PR* partial response

The above results showed that endogenous IFN-λ expression could promote the inflammation in tumor microenvironment (TME), thus we further investigated the characteristics of TME in patients with IFN-λ expression. We first estimated the infiltration abundance of 428 cell types using the DCNet method, and illustrated the immune and stromal cell infiltration levels of each patient with the t-SNE (Figs. 3B, 5A). Then we compared different of immune and stromal cell infiltration abundance between BSE and NSE groups and the results showed that patients with IFN-λ expression had significantly higher levels of cellular infiltration, suggesting that their own complex tumor microenvironment in patients with BSE (p < 0.05, Fig. 5A). Moreover, to further explore the relationship between IFN-λ expression and specific immune cells infiltration levels. We then explored the specific difference of immune cell infiltration levels between the BSE and NSE groups, and examined the correlation between each infiltration cell type and IFN-λ expression (p < 0.05, $\left| {\text{R}} \right|$> 0.19). The results showed that patients with IFN-λ expression had significantly fewer T cell, myeloid conventional dendritic cell and Exhausted CD4 + T cell in TME, but significantly more CD8 + cytotoxic T cell, M1 macrophage, regulatory T cell, CD4 + regulatory T cell, dendritic cell and exhausted T cell (Fig. 5B).

In addition, we showed the extent of immune cell infiltration of the above cell types in patients with BSE and NSE groups using t-SNE. The findings suggest that patients with endogenous IFN-λ expression had more infiltration of immune cells and depletion of T cells compared with NSE patients (Fig. 5C). To further characterize the findings, we obtained the interferon-stimulated gene resistance signature (ISG.RS) and the Interferon-gamma hallmark gene set (IFNG.GS) from the published literature [24], in which IFNG.GS is predominantly expressed by intratumoral immune cells, leading to T cell depletion, and ISG.RS is predominantly expressed in cancer cells. We then compared the expression levels of the ISG.RS and the IFNG.GS in patients with BSE and NSE groups. The results found a significant increase in the expression of ISG.RS and IFNG.GS in patients from the BSE group, indicating that patients with endogenous IFN-λ expression have higher levels of immune cell infiltration and T cell exhaustion compared to patients without endogenous IFN-λ expression, which are consistent with the above findings. (Fig. 5D).

However, patients with endogenous IFN-λ expression did not show a matching survival advantage (Fig. 2E). Therefore, to further reveal the role of the IFN-λ expression in the TME immune regulation. We evaluated the expression levels of genes associated with immune molecular characteristics such as T cell inflammation [25, 26], T cell dysfunction [27] and immune checkpoints. The results showed upregulation expression of T cell dysfunction and inflammation (Fig. 5E) related genes in the BSE patients, indicating that the infiltrating T cells were mainly exhausted T cells and had a high inflammatory environment in patients with endogenous IFN-λ expression. Hence, high gene expression of T-cell dysfunction and inflammatory genes might be responsible for the patients owning a higher level of immune cell infiltration but a lower prognosis in the BSE. Moreover, most of the immune checkpoint genes (except for SIRPA, BTN2A, CD200, HHLA2 and TNFRSF14) were highly expressed in the BSE group (Fig. 5F). Interestingly, immunotherapy response predictors such as PDL1 (CD274), TIGIT and CTLA4 were upregulated in BSE patients, suggesting that patients with endogenous IFN-λ expression were more suitable for immunotherapy. Our study showed that dendritic cell (DC) infiltration was significantly increased in TME of the BSE patients. Dendritic cells are responsible for antigen presentation and activation of naive T cells, are a bridge connecting innate and adaptive immunity, and their activation depending on the high expression level of major histocompatibility complex class (MHC) molecules and adhesion factors [28, 29]. Therefore, we further analyzed the expression levels of MHC molecules in patients with endogenous IFN-λ expression and found that the increased expression of IFN-λ resulted in the comprehensively elevated expression of MHC-I and MHC-II molecules (Fig. 5G). Interestingly, we also observed that the mesenchymal (MES) related gene expression was also upregulated in the BSE group (Fig. 5G). The MES-like states of macrophages and cancer cells may also represent a therapeutic opportunity [30]. So we further investigated whether the expression of IFN-λ affects the therapeutic efficacy of immune checkpoint blockade and analyzed patients with and without IFN-λ expression in the IMvigor210 cohort [31] exhibiting different levels of therapeutic response to anti-PD-L1 blocker. We found patients with IFN-λ expression showed significantly better therapeutic outcomes (Responser/Nonresponer: 31%/69% in the OSE/BSE group and 21%/79% in the NSE group, Fig. 5H).

Overall, our analysis suggests that high gene expression of T-cell dysfunction and strong inflammatory environment might be responsible for the patients owning a higher level of immune cell infiltration but a lower prognosis in the BSE group.

### Patients with endogenous IFN-λ expression exhibited more TP53 mutations and low methylation levels

**Fig. 6:**
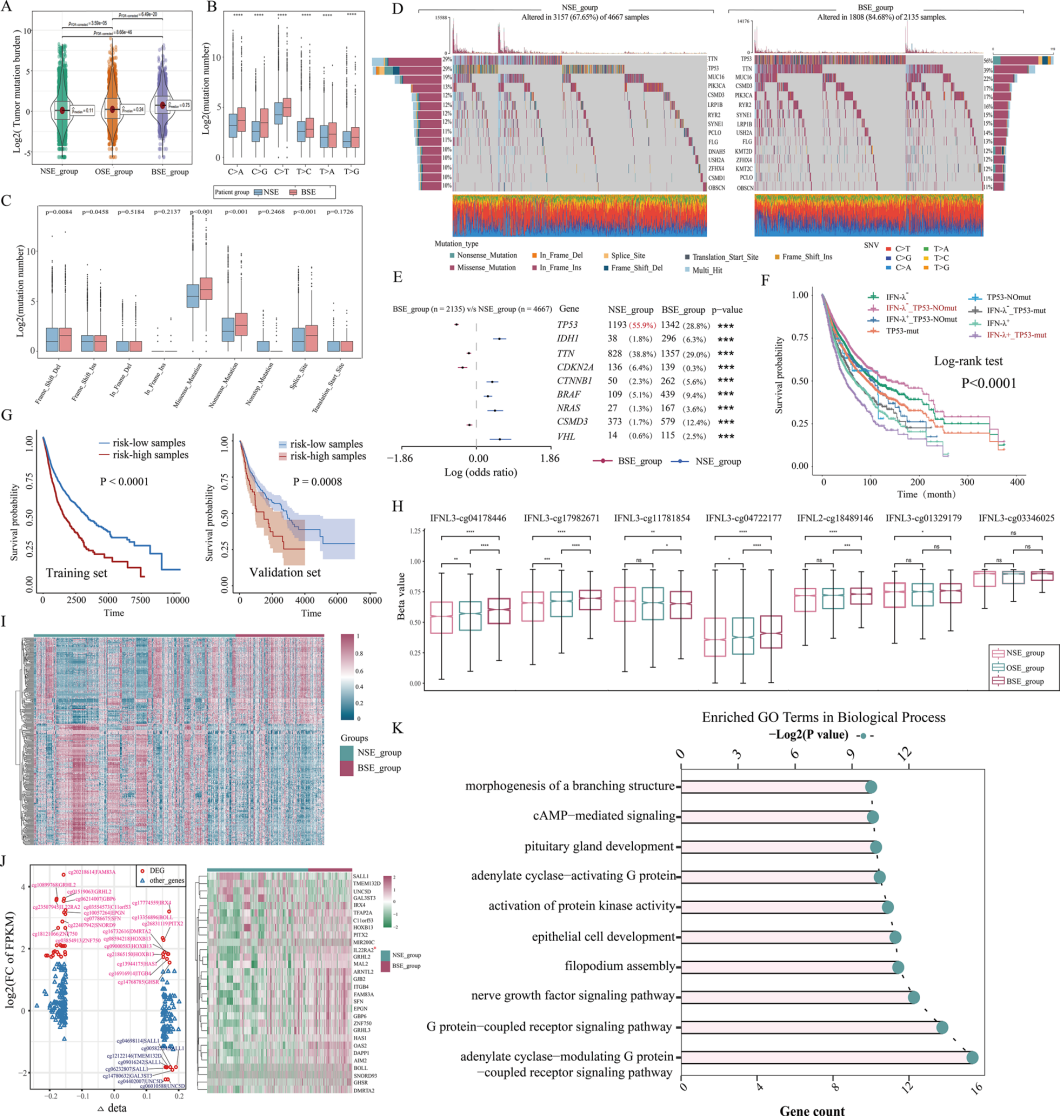
**A** Tumor mutation burden (TMB) among the three IFN-λ subgroups. **B**–**C** Boxplots showing the comparisons of mutation frequencies of **B** SNV and **C** every mutation type classified by effects between the BSE and NSE groups. **D** The mutational landscapes of the BSE and NSE groups. **E** Forest plot displays the top 9 most significantly differentially mutated genes between the BSE and NSE groups. **F** Effects of IFN-λ expression and TP53 mutation status on OS in the training set. **G** Kaplan–Meier curves show the independent relevance between overall survival time and risk scores in the training (left) and validation set (right). **H** Methylation expression levels of IFNL 2 and IFNL 3 among the three IFN-λ expression subgroups. **I** The distribution of DMPs in BSE and NSE groups was exhibited by heatmap. **J** The left plot illustrates the relationships between expression change and DNA methylation level. The nodes in red represent the DEGs with log2FC of FPKM > 1.5 and Δbeta > 0.15. The right plot illustrates the heatmap of these DEGs. **K** The results of GO biological process enrichment analyses on DMP-associated genes

After detecting the IFN-λ expression specificity in the above transcriptome analysis, we further explored whether patients with BSE have unique genetic variation. We compared the mutational load of the three IFN-λ subgroups and found a higher mutational load in the BSE patients group (p < 0.05, Fig. 6A), suggesting that patients with IFN-λ expression had a more complex genomic landscape. Next, different types of somatic mutations, including the single-nucleotide variant (SNV), single-nucleotide polymorphism (SNP), insertion (INS), and deletion (DEL) were analyzed using the R package maftools. Among the detected SNVs, C > T appeared to be the most common mutation in the BSE group (p < 0.05, Fig. 6B). No matter the type of SNV, the mutation numbers in the BSE group were significantly higher than those in the NSE group (p < 0.05, Fig. 6B). Meanwhile, we also found that BSE patients were more inclined to have these three types of mutations, including missense, nonsense and splice-site (p < 0.01, Fig. 6C). Moreover, we compared the top 15 high-frequency mutation gene profiles in BSE and NSE patient groups, and found that more mutations occurred in BSE patients (BSE, 84.68%, NSE, 67.65%), with the high frequency of TP53 gene mutations (BSE, 56%, NSE, 29%, Fig. 6D). We then further analyzed genes with different mutation frequencies between the BSE and NSE groups using the Fisher test. The top of the top nine most significantly mutated genes between the two groups were shown, and found that TP53 occupies the top 1 (Fisher’s exact test, p < 0.01, odds ratio, 0.89, Fig. 6E), suggesting differences in the TP53 mutations in the BSE and NSE groups. These results imply that patients with BSE are more likely to have mutations in TP53, which is an oncogene [32], and that mutations in it cause a poorer prognosis for patients, suggesting one of the reasons for the poor prognosis of the BSE patients. Next, we considered both IFN-λ expression and TP53 mutation status and divided the patients into 8 groups for survival analysis and found that patients with IFN-λ expression and TP53 mutation had the worst overall survival compared with other groups (p < 0.0001, Fig. 6F), suggesting that IFN-λ expression and TP53 mutation may be key features in predicting clinical prognosis.

Then, we partitioned the samples into two groups in the training set (patients with TP53 mutation and IFN-λ expression were defined as high-risk group, patients with TP53 no-mutation and IFN-λ no-expression were defined as low-risk group) for survival analysis, showing a lower OS rate in the high-risk group than in the low-risk group (p < 0.0001, Fig. 6G). The same conclusion was also observed in the validation set (Fig. 6G). Thus, these findings suggest greater discrimination for the combined effects of IFN-λ expression and TP53 mutation in assessing the prognosis risk of patients. In conclusion, patients with endogenous IFN-λ expression have a higher mutational load and the combination of IFN-λ expression and TP53 mutations was a more effective diagnostic factor.

In addition, failure to maintain normal DNA methylation, which consequently increase the susceptibility to triggering tumor formation and deterioration [33, 34]. To explore specific methylation profiles in patients with BSE. We first compared the DNA methylation levels of IFNL2 and IFNL3 among the three IFN-λ subgroups using 450k DNA methylation data from UCSC Xena and found that the BSE patients had a significantly higher levels of methylation, including cg04178446, cg17982671, cg04722177 and cg18489146 probes (Fig. 6H). Then, we examined 436 differential methylation probes (DMPs) in the BSE and NSE groups (Δbeta > 0.15 and FDR < 0.05) and found the BSE patients tended to have hypomethylated positions overall, with 58.48% (255 hypomethylated positions) of the DMPs being hypomethylated (Fig. 6I). Furthermore, we identified a total of 30 DMP-related genes in the BSE and NSE groups using the limma package (log2FC > 1.5, p < 0.05). There were 26 (86.7%) upregulated and 4 (13.3%) downregulated DEGs in the BSE group (Fig. 6J). These results suggest that genomes of BSE patients demonstrate an overall hypomethylation trend and upregulation of DMP-related gene expression. Next, to further investigate the function of these DMP-related genes. The top 10 enriched GO terms of biological processes with the lowest FDRs showed their potential roles in the epithelial cell development and cAMP mediated signaling (Fig. 6K). GSEA of the DMP-associated genes showed that hypomethylated genes were more important contributors to immune-related processes (Additional file 4: Figure S4A), suggesting that the possible effect of hypomethylation on gene overexpression was to trigger an elevated level of immune cell infiltration. Furthermore, to identify IFN-λ-related prognostic signatures from DNA methylated alterations. We obtained 436 DMPs for the above analysis using a univariate cox proportional hazards regression to determine a significant independent effect of 21 methylation on patient overall survival time (p < 0.05), which was used to establish the multivariate Cox proportional risk regression model. Kaplan–Meier survival analyse showed that the high-risk group had a poorer overall survival compared with the low-risk group in the training set (Log-rank test p < 0.001, Additional file 4: Figure S4B). The same results were also obtained in the validation set (Log-rank test p < 0.001, Additional file 4: Figure S4C).

Together, the above results demonstrate that BSE patients with hypomethylation levels, and the identification of IFN-λ-related prognostic signatures from DNA methylated alterations accurately predicts patients’ risk.

### Spatial transcriptomics map revealed CD4 and CD8 cells as the main source of endogenous IFN-λ expression

**Fig. 7:**
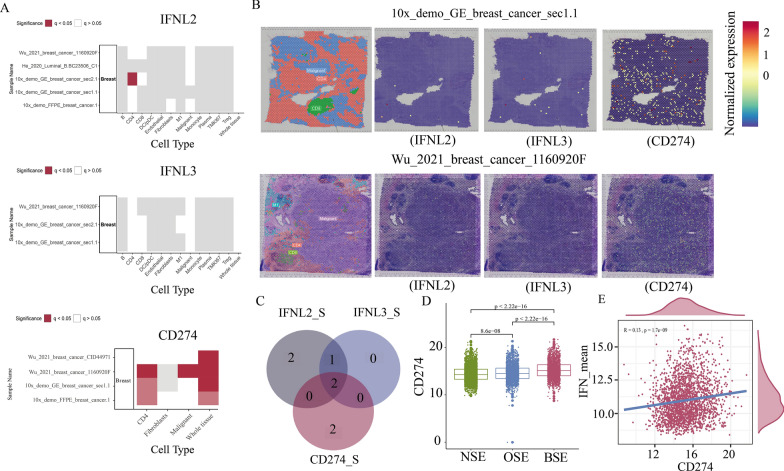
**A** Spatial variability of IFNL2, IFNL3 and CD274 in samples. **B** Normalized spatial expression of genes in three representative samples and annotated cell types. **C** Intersections of samples from three genes spatial variability. **D** Comparisons of the expression of CD274 among the three IFN-λ subgroups. **E** Pearson correlation between IFN-λ expression and CD274 expression

Next, to investigate the cellular origin and spatial variability of IFN-λ2 and IFN-λ3 expression. We examined which cells mainly express IFN-λ2 and IFN-λ3 by using the SOAR database and found significant expression of them in CD4, CD8 and malignant cells (Fig. 7A), which indicates that endogenous IFN-λ expression is mainly secreted by adaptive immune cells such as CD4 + T cells and CD8 + T cells and it has an important role in the immune-editing process. Subsequently, we also checked the spatial distribution of IFN-λ expression in these cells in the two samples (Wu_2021_breast_cancer_1160920F and 10x_demo_GE_breast_cancer_sec1.1) with IFN-λ expression (Fig. 7B), and found a scattered distribution throughout the tumor microenvironment. Since IFN-λ has an important role in the immune-editing process, we also examined the cellular origin (Fig. 7A) and spatial variability (Fig. 7B) of PD-L1 expression. We found PDL1 expression in two samples (Wu_2021_breast_cancer_1160920F and 10x_demo_GE_breast_cancer_sec1.1) in both CD4 cells and malignant cells were also detected (Fig. 7C), indicating that patients with endogenous IFN-λ expression would exhibit significantly high expression of PD-L1. In our previous results, we also found that IFN-λ induced the expression of immune molecules. Whereafter, to further explore the relationship between IFN-λ and PD-L1, we analyzed PD-L1 expression among the three IFN-λ subgroups and found a significant upregulation of PD-L1 expression in the BSE group (p < 0.001 Fig. 7D). Then, We found a significant positive correlation between PD-L1 (CD274) and IFN-λ expression using the pearson correlation analysis (p < 0.001 Fig. 7E). In conclusion, our analysis revealed that the main source of endogenous IFN-λ expression might be CD4 and CD8 cell production, and that patients with endogenous IFN-λ expression showed high expression of PD-L1, suggesting a potential response to anti-PD-1/L1 immunotherapy.

## Discussion

Increasing evidence demonstrated that IFN-λ can also promote oncogenesis, indicating its dual role in cancer [35]. However, the dark side of endogenous IFN-λ remain unclear in comprehensive genetic and transcriptome landscape across cancer types, suggesting the need for comprehensive review analysis. In this study, we explored the expression and prognostic value of IFN-λ in human tumors, as well as the mechanisms of tumor progression.

We performed a comprehensive study and analysis of the expression pattern of endogenous IFN-λ in cancer samples from TCGA. We found that IFN-λ was specifically expressed in cancer patients and patients with endogenous IFN-λ expression exhibited a trend toward poorer survival in many tumor types and that IFN-λ also acts as a cancer promoter [36]. However, the result of poorer survival in patients with endogenous IFN-λ expression was not shown in a small number of tumor types, which could be explained by the low endogenous expression levels of IFN-λ and consequently the weak driving effect of patients with some tumor types. Moreover, IFN-λ2 and IFN-λ3 could be used as independent predictors of survival outcome (Additional file 5: Figure S5B).

Moreover, the transcriptome of IFN-λ-related genes exhibited greatly perturbed in cancer. We identified IFN-λ-related genes (e.g. STAT1, STAT2, STAT3, IL24, IL10, IFNL1, IL26, IL10RB, and IFNL1) from different levels, including receptor-ligand interactions, protein–protein interactions and transcriptional regulation. We discovered that these IFN-λ-related genes work together with IFN-λ to influence the inflammatory response and the JAK-STAT pathway to promote cancer progression. Several studies have also shown that the JAK/STAT activation is observed to be associated with a poor prognosis in many tumor types [37–39]. Furthermore, STAT is extensively associated with cancer cell survival, immunosuppression and persistent inflammation in the tumor microenvironment [40]. These results indicated that IFN-λ plays an important role in cancer immunology [41, 42].

Subsequently, we evaluated the effect of copy number alterations on the expressions of IFN-λ genes. The results showed that endogenous IFN-λ expression may be mainly caused by copy number amplification of IFNL2 and IFNL3 genes in tumor patients. Consequently, IFN-λ promotes the activity of transcription factors STAT1, STAT2, and STAT3 by influencing the JAK-STAT signaling pathway, leading to the dysregulation of downstream gene expression (Additional file 5: Figure S5A), such as matrix metalloproteinases (MMPs), including MMP1, MMP3, MMP7, MMP10, and MMP13 genes, which are almost universally upregulated in cancer [20].

Our study also evaluated differences in somatic alterations and DNA methylation in patients with and without endogenous IFN-λ expression. Patients with endogenous IFN-λ expression exhibited a higher mutation load and more TP53 gene mutations. The results also found that patients with TP53 gene mutation and IFN-λ expression had a worse prognosis compared with patients with TP53 no-mutation and IFN-λ no-expression, implying that the combined effect of TP53 mutation and IFN-λ expression could be used as prognostic factor (Figure S5C). In addition, it has been shown that the DNA methylation was involved in gene regulation [43]. Therefore, our study continue to compare the differences in DNA methylation levels between patients with and without endogenous IFN-λ expression. The epigenetic alterations suggested that BSE patients exhibited unique hypomethylation patterns (Additional file 5: Figure S5D). The functional enrichment analysis of several IFN-λ-related genes with differentially methylated promoters indicated that they are involved in epithelial cell development, cAMP-mediated signaling and further promoting cancer progression.

Furthermore, our study has found that the patients with endogenous IFN-λ expression show unique tumor microenvironments and its potential role for immunotherapy (Additional file 5: Figure S5E). The results exhibited a higher levels of immune cell infiltration in patients with endogenous IFN-λ expression. However, patients with endogenous IFN-λ expression with higher immune infiltration did not show a matching survival advantage. So, we further explored and discovered an upregulation of gene expression related to T-cell dysfunction in the patients with endogenous IFN-λ expression. Thus, even though the BSE patients have higher levels of immune cell infiltration, the higher intensity of T cell dysfunction resulted in poorer patient survival [44, 45].

Moreover, the expression of IFN-λ was positively correlated with M1 macrophage, exhausted T cell, NKT cells and CD8 + cytotoxic T cells infiltrations. It has been reported that the cytotoxicity and phagocytosis of macrophages, as well as the secretion of pro-inflammatory cytokines and interferon-stimulated genes is stimulated by IFN-λ expression [46, 47]. Here, the patients with endogenous IFN-λ expression exhibited higher M1 macrophage infiltration, suggesting that IFN-λ may act as an effective adjuvant in promoting the immune evasion function of macrophages. Due to its unique tumor microenvironment characteristics, we further investigated the therapeutic efficacy of immune checkpoint blockade in patients with and without IFN-λ expression. It was also found that patients with IFN-λ expression had higher expression of immune checkpoint genes, which maybe respond well to immunotherapy.

Our work incorporated multi-omics data to propose a systematic approach to reveal the shadowy side of cancer IFN-λ and to explore the clinical and immunological features of endogenously expressed IFN-λ in tumor samples as well as to uncover the genomic, transcriptional and epigenetic mechanisms mediating IFN-λ-specific expression. In summary, we show the expression of endogenous IFN-λ on the dark side of cancer and provide a fundamental resource for further cancer discovery and therapeutic exploration.

## Conclusions

In summary, the analysis of multi-omics supported the important roles of IFN-λ in tumorigenesis. The patients with endogenous IFN-λ expression are significantly associated with poor prognosis. Most importantly, amplification of IFN-λ copy number variation drove endogenous IFN-λ-specific expression in patients. This work provided a comprehensive overview and analysis of genetic landscape of endogenous IFN-λ across cancer types, which will provide comprehensive insights into the dark ‘‘tumor-promoting’’ side effect of endogenous IFN-λ and shed light on future development of therapeutic targets.

### Supplementary Information

**Additional file 1: Figure S1.** **A** CDF curve with k = 2–10 using the K-means method. **B** Delta area plot showed the relative change in area under the CDF curve.

**Additional file 2: Figure S2.** A Interaction of the enriched pathways. The size represents the number of genes.

**Additional file 3: Figure S3.** **A** Univariate and multivariate analyses of the clinical characteristics and risk score with the OS in validation set.

**Additional file 4: Figure S4. A** Gene set enrichment analysis (GSEA) of the DMP-associated genes showed that the significant enrichment in immune-related processes. **B** Kaplan-Meier curves show the independent relevance between overall survival time and risk scores in the training set **C** Kaplan-Meier curves show the independent relevance between overall survival time and risk scores in the validation set (right).

**Additional file 5:  Figure S5.** **A** The endogenous tumor-specific expression of IFN-λ affects the JAK-STAT signaling pathway, activating the transcription factors STAT, and inducing downstream signaling cascades. **B** Kaplan-Meier estimates of overall survival for the IFN_exp and the rest group in the training and validation cohort. **C** The patients with endogenous IFN-λ expression exhibited more TP53 mutations and low methylation levels. **D** Characteristics of the cancer microenvironment of endogenous IFN-λ and its potential role for immunotherapy.

## Data Availability

The original contributions presented in the study are included in the article. Further inquiries can be directed to the corresponding author.

## References

[1] Kotenko SV, Gallagher G, Baurin VV (2003). IFN-lambdas mediate antiviral protection through a distinct class II cytokine receptor complex. Nat Immunol.

[2] Sheppard P, Kindsvogel W, Xu W (2003). IL-28, IL-29 and their class II cytokine receptor IL-28R. Nat Immunol.

[3] Tezuka Y, Endo S, Matsui A (2012). Potential anti-tumor effect of IFN-lambda2 (IL-28A) against human lung cancer cells. Lung Cancer.

[4] Li Q, Kawamura K, Ma G (2010). Interferon-lambda induces G1 phase arrest or apoptosis in oesophageal carcinoma cells and produces anti-tumour effects in combination with anti-cancer agents. Eur J Cancer.

[5] Lasfar A, Lewis-Antes A, Smirnov SV (2006). Characterization of the mouse IFN-lambda ligand-receptor system: IFN-lambdas exhibit antitumor activity against B16 melanoma. Cancer Res.

[6] Burkart C, Arimoto K, Tang T (2013). Usp18 deficient mammary epithelial cells create an antitumour environment driven by hypersensitivity to IFN-lambda and elevated secretion of Cxcl10. EMBO Mol Med.

[7] Lee SJ, Lee EJ, Kim SK (2012). Identification of pro-inflammatory cytokines associated with muscle invasive bladder cancer; the roles of IL-5, IL-20, and IL-28A. PLoS ONE.

[8] Mucha J, Majchrzak K, Taciak B (2014). MDSCs mediate angiogenesis and predispose canine mammary tumor cells for metastasis via IL-28/IL-28RA (IFN-lambda) signaling. PLoS ONE.

[9] Pingwara R, Witt-Jurkowska K, Ulewicz K (2017). Interferon lambda 2 promotes mammary tumor metastasis via angiogenesis extension and stimulation of cancer cell migration. J Physiol Pharmacol.

[10] Wilkerson MD, Hayes DN (2010). Consensusclusterplus: a class discovery tool with confidence assessments and item tracking. Bioinformatics.

[11] Wang X, Wang H, Liu D (2022). Deep learning using bulk RNA-seq data expands cell landscape identification in tumor microenvironment. Oncoimmunology.

[12] Koboldt DC, Zhang Q, Larson DE (2012). VarScan 2: somatic mutation and copy number alteration discovery in cancer by exome sequencing. Genome Res.

[13] Mayakonda A, Lin DC, Assenov Y (2018). Maftools: efficient and comprehensive analysis of somatic variants in cancer. Genome Res.

[14] Hanzelmann S, Castelo R, Guinney J (2013). GSVA: gene set variation analysis for microarray and RNA-seq data. BMC Bioinform.

[15] Ritchie ME, Phipson B, Wu D (2015). limma powers differential expression analyses for RNA-sequencing and microarray studies. Nucleic Acids Res.

[16] Szklarczyk D, Gable AL, Lyon D (2019). STRING v11: protein-protein association networks with increased coverage, supporting functional discovery in genome-wide experimental datasets. Nucleic Acids Res.

[17] Robin X, Turck N, Hainard A (2011). pROC: an open-source package for R and S+ to analyze and compare ROC curves. BMC Bioinform.

[18] Yu G, Wang LG, Han Y (2012). clusterProfiler: an R package for comparing biological themes among gene clusters. OMICS.

[19] Johnson DE, O'Keefe RA, Grandis JR (2018). Targeting the IL-6/JAK/STAT3 signalling axis in cancer. Nat Rev Clin Oncol.

[20] Gobin E, Bagwell K, Wagner J (2019). A pan-cancer perspective of matrix metalloproteases (MMP) gene expression profile and their diagnostic/prognostic potential. BMC Cancer.

[21] Tokunaga R, Naseem M, Lo JH (2019). B cell and B cell-related pathways for novel cancer treatments. Cancer Treat Rev.

[22] Khalid Z, Huan M (2022). Identification of novel therapeutic candidates against SARS-CoV-2 infections: an application of RNA sequencing toward mrna based nanotherapeutics. Front Microbiol.

[23] Bandiera E, Zanotti L, Bignotti E (2009). Human kallikrein 5: an interesting novel biomarker in ovarian cancer patients that elicits humoral response. Int J Gynecol Cancer.

[24] Benci JL, Johnson LR, Choa R (2019). Opposing functions of interferon coordinate adaptive and innate immune responses to cancer immune checkpoint blockade. Cell..

[25] Jiang P, Gu S, Pan D (2018). Signatures of T cell dysfunction and exclusion predict cancer immunotherapy response [J]. Nat Med.

[26] Zhao Y, Shao Q, Peng G (2020). Exhaustion and senescence: two crucial dysfunctional states of T cells in the tumor microenvironment. Cell Mol Immunol.

[27] Bao R, Stapor D, Luke JJ (2020). Molecular correlates and therapeutic targets in T cell-inflamed versus non-T cell-inflamed tumors across cancer types. Genome Med.

[28] Hemann EA, Green R, Turnbull JB (2019). Interferon-lambda modulates dendritic cells to facilitate T cell immunity during infection with influenza a virus. Nat Immunol.

[29] Johnson DB, Estrada MV, Salgado R (2016). Melanoma-specific MHC-II expression represents a tumour-autonomous phenotype and predicts response to anti-PD-1/PD-L1 therapy. Nat Commun.

[30] Hara T, Chanoch-Myers R, Mathewson ND (2021). Interactions between cancer cells and immune cells drive transitions to mesenchymal-like states in glioblastoma. Cancer Cell.

[31] Mariathasan S, Turley SJ, Nickles D (2018). TGFbeta attenuates tumour response to PD-L1 blockade by contributing to exclusion of T cells. Nature.

[32] Bernard E, Nannya Y, Hasserjian RP (2020). Implications of TP53 allelic state for genome stability, clinical presentation and outcomes in myelodysplastic syndromes. Nat Med.

[33] Koch A, Joosten SC, Feng Z (2018). Analysis of DNA methylation in cancer: location revisited. Nat Rev Clin Oncol.

[34] Soozangar N, Sadeghi MR, Jeddi F (2018). Comparison of genome-wide analysis techniques to DNA methylation analysis in human cancer. J Cell Physiol.

[35] Lasfar A, Zloza A, Silk AW (2019). Interferon lambda: toward a dual role in cancer. J Interferon Cytokine Res.

[36] Souza-Fonseca-guimaraes F, Young A, Mittal D (2015). NK cells require IL-28R for optimal in vivo activity. Proc Natl Acad Sci USA.

[37] Hu X, Li J, Fu M (2021). The JAK/STAT ignaling pathway: from bench to clinic. Signal Transduct Target Ther.

[38] Shao F, Pang X, Baeg GH (2021). Targeting the JAK/STAT signaling pathway for breast cancer. Curr Med Chem.

[39] Thomas SJ, Snowden JA, Zeidler MP (2015). The role of JAK/STAT signalling in the pathogenesis, prognosis and treatment of solid tumours. Br J Cancer.

[40] Owen KL, Brockwell NK, Parker BS (2019). JAK-STAT signaling: a double-edged sword of immune regulation and cancer progression. Cancers.

[41] Lasfar A, Gogas H, Zloza A (2016). IFN-lambda cancer immunotherapy: new kid on the block. Immunotherapy.

[42] Manivasagam S, Klein RS (2021). Type III interferons: emerging roles in autoimmunity. Front Immunol.

[43] Ghoneim HE, Fan Y, Moustaki A (2017). De novo epigenetic programs inhibit PD-1 blockade-mediated T cell rejuvenation. Cell.

[44] Thommen DS, Schumacher TN (2018). T cell dysfunction in cancer. Cancer Cell.

[45] Xia A, Zhang Y, Xu J (2019). T cell dysfunction in cancer immunity and immunotherapy. Front Immunol.

[46] Read SA, Wijaya R, Ramezani-Moghadam M (2019). Macrophage coordination of the interferon lambda immune response. Front Immunol.

[47] Yunna C, Mengru H, Lei W (2020). Macrophage M1/M2 polarization. Eur J Pharmacol.

