# Supplementary figures and images for: Portraying the dark side of endogenous IFN-λ for promoting cancer progression and immunoevasion in pan-cancer
Source: J Transl Med. 2023 Sep 11;21:615. doi: 10.1186/s12967-023-04453-4 (PMC10494394; doi:10.1186/s12967-023-04453-4)

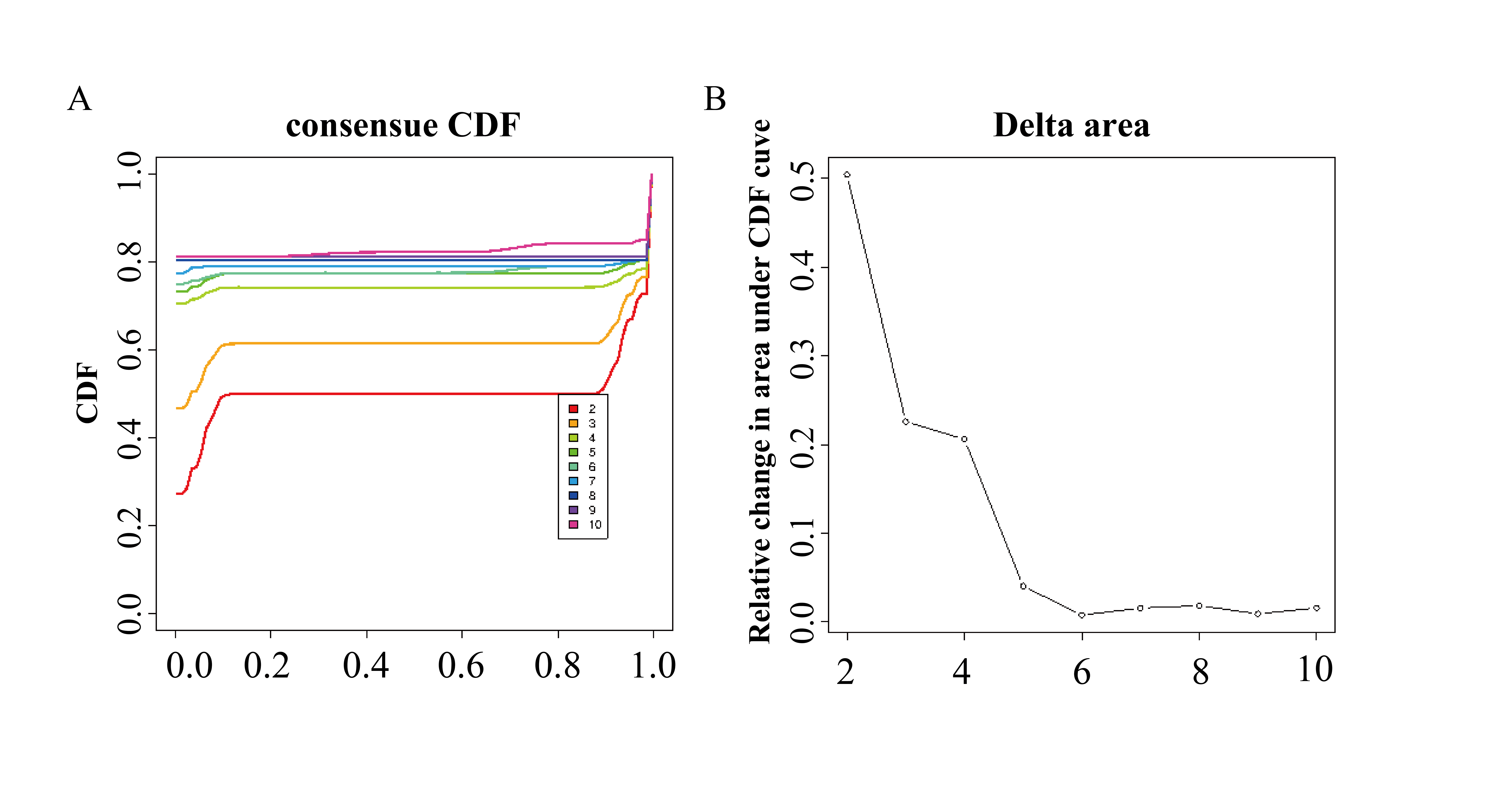

Supplement: Supplementary file 1 — Additional file 1: Figure S1. A CDF curve with k = 2–10 using the K-means method. B Delta area plot showed the relative change in area under the CDF curve. [file 12967_2023_4453_MOESM1_ESM.png]

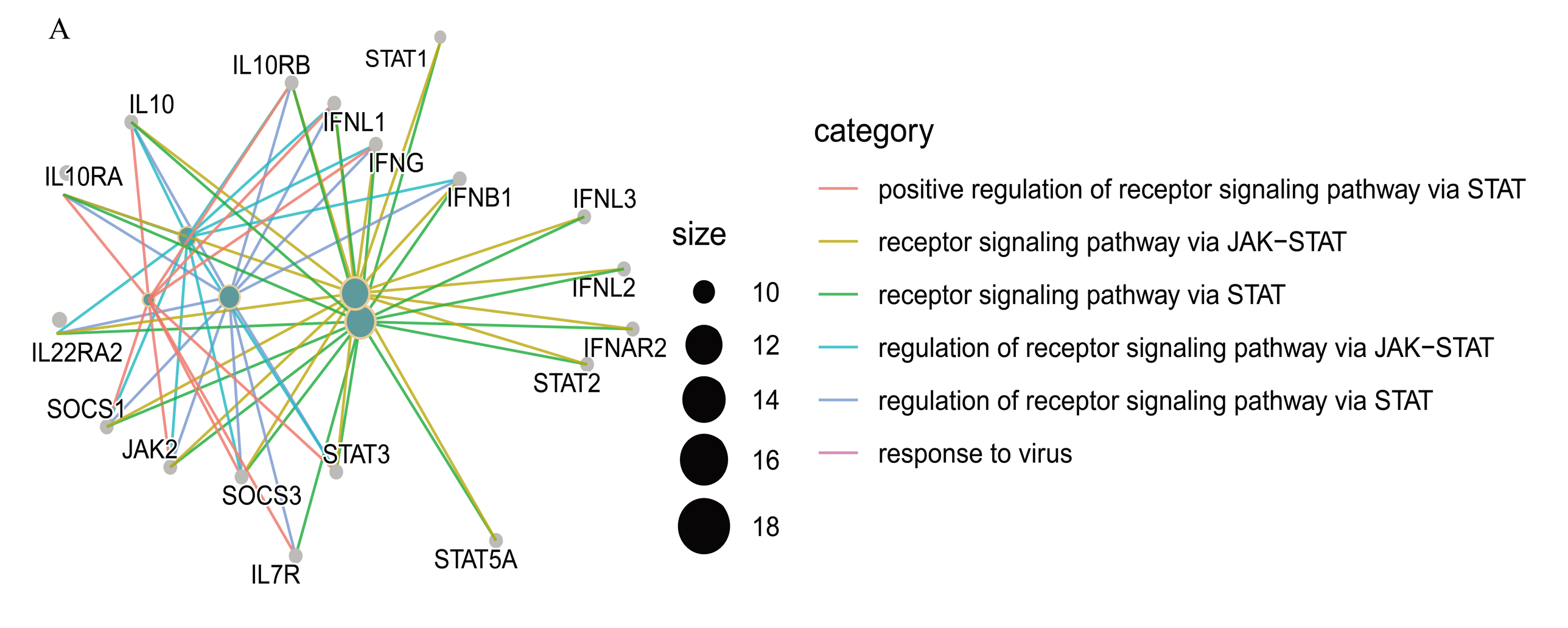

Supplement: Supplementary file 2 — Additional file 2: Figure S2. A Interaction of the enriched pathways. The size represents the number of genes. [file 12967_2023_4453_MOESM2_ESM.png]

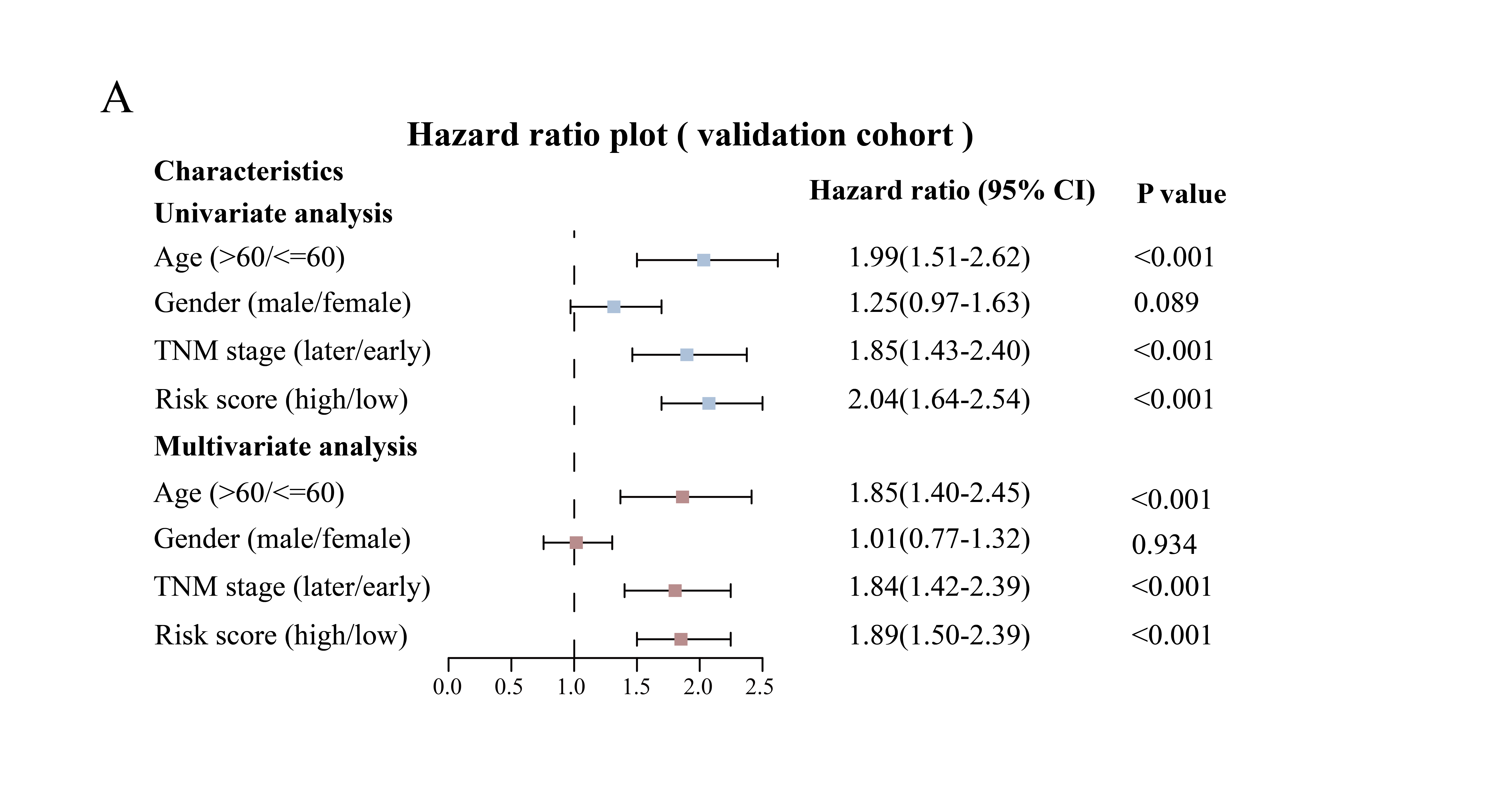

Supplement: Supplementary file 3 — Additional file 3: Figure S3. A Univariate and multivariate analyses of the clinical characteristics and risk score with the OS in validation set. [file 12967_2023_4453_MOESM3_ESM.png]

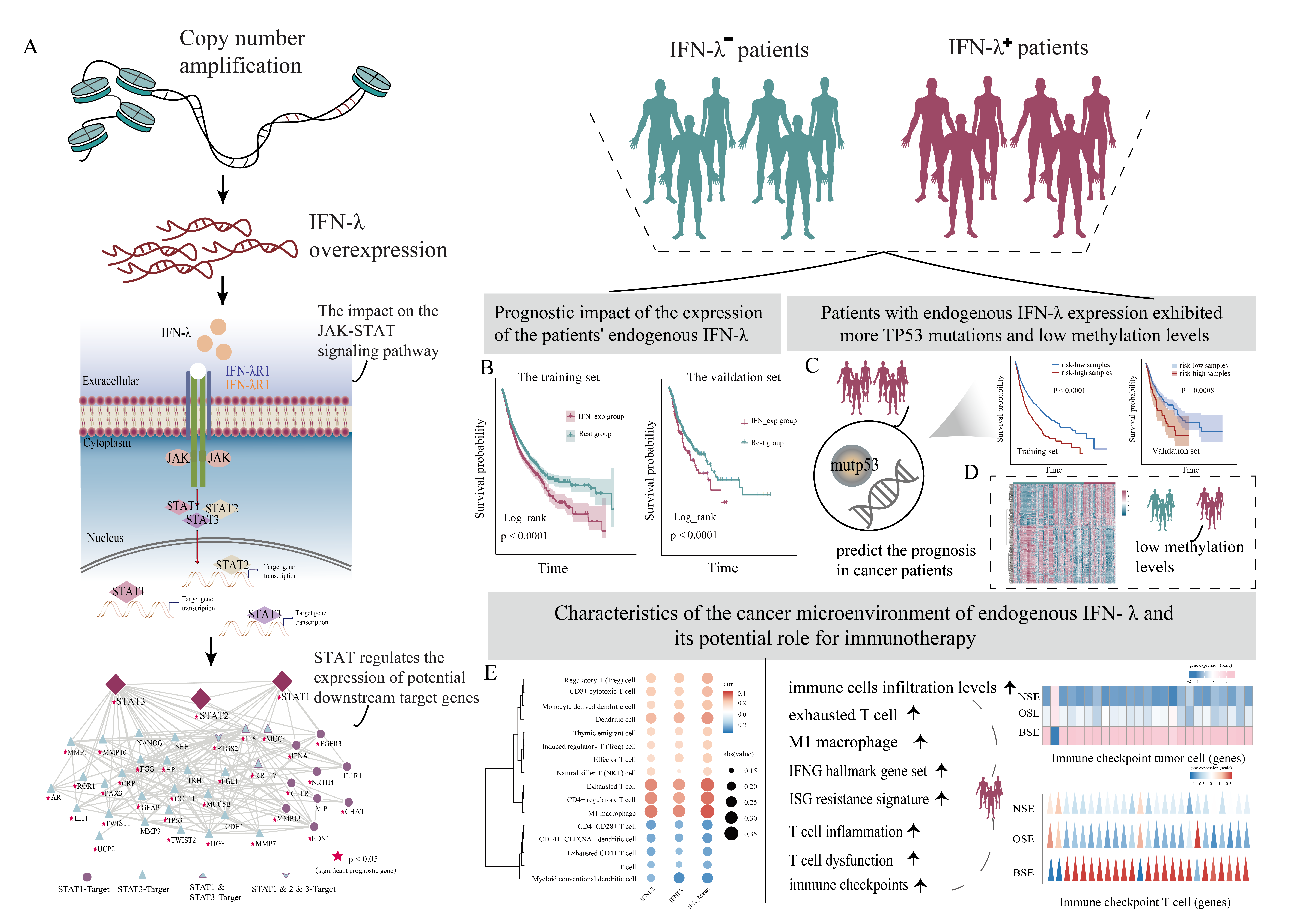

Supplement: Supplementary file 5 — Additional file 5: Figure S5. A The endogenous tumor-specific expression of IFN-λ affects the JAK-STAT signaling pathway, activating the transcription factors STAT, and inducing downstream signaling cascades. B Kaplan-Meier estimates of overall survival for the IFN_exp and the rest group in the training and validation cohort. C The patients with endogenous IFN-λ expression exhibited more TP53 mutations and low methylation levels. D Characteristics of the cancer microenvironment of endogenous IFN-λ and its potential role for immunotherapy. [file 12967_2023_4453_MOESM5_ESM.png]
